# Persistent Neighborhood Poverty and Breast Cancer Outcomes

**DOI:** 10.1001/jamanetworkopen.2024.27755

**Published:** 2024-08-29

**Authors:** J. C. Chen, Demond Handley, Mohamed I. Elsaid, James L. Fisher, Jesse J. Plascak, Lisa Anderson, Carolyn Tsung, Joal Beane, Timothy M. Pawlik, Samilia Obeng-Gyasi

**Affiliations:** 1 Division of Surgical Oncology, Department of Surgery, The Ohio State University Wexner Medical Center and James Cancer Hospital, Columbus; 2 Department of Biomedical Informatics, College of Medicine, The Ohio State University, Columbus; 3 Center for Biostatistics, College of Medicine, The Ohio State University, Columbus; 4 Division of Medical Oncology, Department of Internal Medicine, College of Medicine, The Ohio State University, Columbus; 5 The Ohio State University College of Medicine, Columbus; 6 James Cancer Hospital and Solove Research Institute, Columbus; 7 Division of Cancer Prevention and Control, Department of Internal Medicine, College of Medicine, The Ohio State University, Columbus; 8 Washington University in St Louis, St Louis, Missouri

## Abstract

**IMPORTANCE:**

Patients with breast cancer residing in socioeconomically disadvantaged communities often face poorer outcomes (eg, mortality) compared with individuals living in neighborhoods without persistent poverty.

**OBJECTIVE:**

To examine persistent neighborhood poverty and breast tumor characteristics, surgical treatment, and mortality.

**DESIGN, Setting, and Participants:**

A retrospective cohort analysis of women aged 18 years or older diagnosed with stage I to III breast cancer between January 1, 2010, and December 31, 2018, and followed up until December 31, 2020, was conducted. Data were obtained from the Surveillance, Epidemiology, and End Results Program, and data analysis was performed from August 2023 to March 2024.

**EXPOSURE:**

Residence in areas affected by persistent poverty is defined as a condition where 20% or more of the population has lived below the poverty level for approximately 30 years.

**MAIN OUTCOME AND MEASURES:**

All-cause and breast cancer–specific mortality.

**RESULTS:**

Among 312 145 patients (mean [SD] age, 61.9 [13.3] years), 20 007 (6.4%) lived in a CT with persistent poverty. Compared with individuals living in areas without persistent poverty, patients residing in persistently impoverished CTs were more likely to identify as Black (8735 of 20 007 [43.7%] vs 29 588 of 292 138 [10.1%]; *P* < .001) or Hispanic (2605 of 20 007 [13.0%] vs 23 792 of 292 138 [8.1%]; *P* < .001), and present with more-aggressive tumor characteristics, including higher grade disease, triple-negative breast cancer, and advanced stage. A higher proportion of patients residing in areas with persistent poverty underwent mastectomy and axillary lymph node dissection. Living in a persistently impoverished CT was associated with a higher risk of breast cancer–specific (adjusted hazard ratio [AHR], 1.10; 95% CI, 1.03-1.17) and all-cause (AHR, 1.13; 95% CI, 1.08-1.18) mortality. As early as 3 years following diagnosis, mortality risks diverged for both breast cancer–specific (rate ratio [RR], 1.80; 95% CI, 1.68-1.92) and all-cause (RR, 1.62; 95% CI, 1.56-1.70) mortality.

**CONCLUSIONS AND RELEVANCE:**

In this cohort study of women aged 18 years or older diagnosed with stage I to III breast cancer between 2010 and 2018, living in neighborhoods characterized by persistent poverty had implications on tumor characteristics, surgical management, and mortality.

## Key Points

**Question:**

Is residential persistent poverty associated with breast cancer characteristics, treatment, and mortality?

**Findings:**

In this cohort study analyzing 312 145 patients in the Surveillance, Epidemiology, and End Results Program, individuals residing in areas with persistent poverty experienced more-aggressive tumor characteristics and underwent higher rates of mastectomy and axillary lymph node dissection compared with individuals residing in areas without persistent poverty. In addition, impoverished individuals had a 10% increased risk of breast cancer–specific mortality and a 13% increased risk of all-cause mortality.

**Meaning:**

The findings of this study suggest that residing in persistently impoverished neighborhoods is associated with poor tumor characteristics and increased mortality.

## Teaser

This cohort study examines tumor characteristics, treatment, and mortality in women with breast cancer living in persistently impoverished census tracts.

## Introduction

The social and built environments in which people live have well-established major implications for health behaviors, access to resources, and consequential health outcomes.^1^ Among patients with breast cancer, neighborhood factors, such as socioeconomic status and racial residential segregation, have been associated with higher breast cancer incidence and more-aggressive tumor characteristics (ie, molecular subtype and disease burden) at presentation.^2,3,4,5^ Neighborhood racial composition, segregation, and income have also been associated with breast cancer–specific and all-cause mortality.^6^

Persistent poverty refers to geographic areas with poverty rates greater than or equal to 20% over the last 30 years.^7^ Persistent poverty, in contrast to transitory or chronic poverty, refers to geographic locations rather than individuals and families with high poverty rates for an extended time.^7,8^ Areas of persistent poverty are characterized by systemic and structural decay, as property values yielding low investment returns disincentivize property owners from spending money to maintain and/or improve property.^7,8^ Basic necessities, such as public services (eg, utilities, public transportation, law enforcement), food accessibility, education, health care services, support services, and social programs, are affected. In addition, social and safety networks decline, ultimately leading to intergenerational poverty.^7,8,9^

Recent studies have highlighted how people living in geographic areas with persistent poverty experience higher cancer mortality rates, over and above the risk associated with current poverty.^10,11^ Patients with breast cancer living in areas of persistent poverty may have more-advanced disease at diagnosis and differences in treatment; however, additional research is needed.^12^ The purpose of this study was to evaluate persistent poverty over time at the census tract (CT) level, which more closely resembles neighborhoods, and breast tumor characteristics, treatment, and mortality.

## Methods

### Data Source

The Surveillance, Epidemiology, and End Results (SEER) Program currently represents nearly 50% of the US population with cancer.^13^ A collection of 18 SEER Program registries was used to identify women aged 18 years or older with stage I to III breast cancer diagnosed from January 1, 2010, to December 31, 2018 (eFigure 1 in Supplement 1).^14^ This specialized dataset contains information about CT-based measures of rural-urban status and persistent poverty. Patients with missing exposure (ie, CT-persistent poverty), survival, sociodemographic, or clinical data were excluded (203 054 patients of the original 587 107 breast cancer cohort [34.6%]). Additionally, patients with American Joint Committee on Cancer stage IV or SEER summary stage representing distant disease and those who received local tumor surgical treatment were excluded. The Strengthening the Reporting of Observational Studies in Epidemiology (STROBE) reporting guideline for cohort studies was followed.^15^ This study was exempt from institutional review and informed consent was waived according to the Common Rule (45 CFR §46), as the analysis of deidentified, publicly available data is not considered human participant research.

### Sociodemographic, Clinical, and Treatment Characteristics

Sociodemographic variables collected included age, race and ethnicity (Black, Hispanic, White, Other), marital status (married/partnered, single, separated/divorced, and widowed), rural-urban status, and Yost Index.^16^ Due to persistent racial and ethnic disparities in breast cancer outcomes, race and ethnicity were included in the study analysis. Racial and ethnic categories were abstracted from medical records, hindering our ability to identify the method of classification, as the initial collection of this information varies by health care facility and practitioner.^17^ American Indian or Alaska Native, Asian or Pacific Islander, and Other were collapsed into an Other category given small sample sizes. Racial categories in SEER are a social construct as genetic ancestry is not available in the SEER database.^18^ Rural-urban status was based on the US Department of Agriculture’s Rural Urban Commuting Area codes (rural, urban, and unknown).^19^

Relevant clinical factors were cancer grade, molecular subtype, and SEER summary (localized, regional) stages. Molecular subtypes were categorized based on hormone receptor (HR) status (estrogen or progesterone) and presence of human epidermal growth factor receptor 2 (*ERBB2*, formerly known as *HER2*): HR^+^/*ERBB2*^+^, HR^+^/*ERBB2*^−^, HR^−^/*ERBB2*^+^, and HR^−^/*ERBB2*^−^.

Receipt of chemotherapy (yes, no/unknown), radiotherapy (yes, no/unknown, refused), breast surgery type (lumpectomy, mastectomy), axillary surgery type (sentinel lymph node biopsy [SLNB] vs axillary lymph node dissection [ALND]), and breast reconstruction (yes, no, unknown) were recorded.

### Exposure

Persistent poverty was defined as CTs in which 20% or more of the population lived below the poverty level for at least 30 years based on 1990 and 2000 decennial censuses and 2007-2011 and 2015-2019 American Community Survey 5-year estimates.^19^ Census tracts are subdivisions of a county with an average of 4000 residents that allow for more precise estimates of persistent poverty.^8^ Use of CTs recently noted an additional 3% of the total US population was living in persistent poverty who otherwise would have been missed with the use of county-level measures.^8^ Patients were categorized as residing vs not residing in a persistently impoverished CT.

### Outcomes

The primary outcomes were all-cause mortality and breast cancer–specific mortality. The follow-up period was from date of diagnosis to the event date, defined as the date of death for patients who died or either the date of last follow-up or the end of the study for patients presumed to be alive (December 31, 2020), whichever came first. Patients who did not experience the event were censored at the date of the last follow-up or at the end of the study, whichever occurred earlier. For breast cancer–specific mortality, patients were censored on the non–breast cancer event date if the cause of death was due to causes other than breast cancer. Secondary outcomes included breast surgery type, axillary surgery type, and receipt of breast reconstruction.

### Statistical Analysis

Data analysis was performed from August 2023 to March 2024. Sociodemographic, clinical, and treatment characteristics were summarized using means (SDs) for continuous variables and frequencies and percentages for categorical variables. Differences in sociodemographic, clinical, and treatment characteristics were compared between patients living in CTs with and without persistent poverty, using *t* tests for continuous variables and χ^2^ tests for categorical variables. With 2-sided, unpaired testing, *P* < .05 was considered statistically significant.

Differences in all-cause and breast cancer–specific mortality were assessed using Kaplan-Meier analysis. Crude and adjusted Cox proportional hazards regression models were fitted to assess the association between persistent poverty and all-cause mortality and between breast cancer–specific mortality and persistent poverty. Proportional hazards assumptions were confirmed visually using the negative-log curve of the survival distribution function and by including a time-dependent covariate in the regression model. Final models were adjusted for age, race and ethnicity, marital status, Rural Urban Commuting Area code, pathologic grade, SEER stage, molecular subtype, receipt of chemotherapy and radiotherapy, and breast and axillary surgery type.

All-cause mortality and breast cancer–specific mortality rates were estimated at 3, 6, and 9 years of follow-up by dividing the number of observed deaths by the total person-years of follow-up for patients living in CTs with and without persistent poverty. Mortality rate differences were calculated by subtracting the mortality rate for patients living in CTs without persistent poverty from mortality rates for patients living in CTs with persistent poverty. Crude rate ratios (RRs) were calculated by dividing the mortality rate for patients living in CTs with persistent poverty by mortality rates for patients living in CTs without persistent poverty. Analysis was conducted with SAS, version 9.4 software (SAS Institute).

## Results

### Patient Population

A total of 312 145 patients (mean [SD] age, 61.9 [13.3] years) met the inclusion criteria, of whom 20 007 (6.4%) lived in persistently impoverished CTs (Table 1). A larger proportion of patients living in persistently impoverished areas were categorized as Black (8735 of 20 007 [43.7%] vs 29 588 of 292 138 [10.1%]; *P* < .001) or Hispanic (2605 of 20 007 [13.0%] vs 23 792 of 292 138 [8.1%]; *P* < .001), without a partner (12 121 of 20 007 [60.6%] vs 117 324 of 292 138 [40.2%]; *P* < .001), and lived in CTs designated as rural (5358 of 20 007 [26.8%] vs 26 129 of 292 138 [8.9%]; *P* < .001). Additionally, a larger proportion of patients living in persistently impoverished CTs presented with more-aggressive tumor characteristics, including higher cancer grade (grade 3, 7365 of 20 007 [36.9%] vs 85 609 of 292 138 [29.3%]; *P* < .001), triple-negative disease (3124 of 20 007 [15.6%] vs 29 496 of 292 138 [10.1%]; *P* < .001), and greater stage of disease (SEER regional, 6712 of 20 007 [33.5%] vs 81 767 of 292 138 [28.0%]; *P* < .001). Patients living in persistently impoverished areas were more likely to receive chemotherapy (8504 of 20 007 [42.5%] vs 108 163 of 292 138 [37.0%]; *P* < .001) and undergo mastectomies (9013 of 20 007 [45.0%] vs 116 134 of 292 138 [39.8%]; *P* < .001) and ALND (4319 of 20 007 [21.6%] vs 44 616 to 292 138 [15.3%]; *P* < .001). Furthermore, patients from persistently impoverished areas were less likely to undergo reconstruction (10 590 of 20 007 [52.9%] vs 124 659 of 292 138 [42.7%]; *P* < .001) after mastectomy than individuals living in areas without persistent poverty. Patients living in persistently impoverished areas were also less likely to receive radiotherapy (9496 of 20 007 [47.5%] vs 122 459 of 292 138 [41.9%]; *P* < .001) compared with patients living in CTs without persistent poverty.

**Table 1.** Sociodemographic, Clinical, and Treatment Characteristics by Residence in Persistently Impoverished CTs

Patient characteristic	No. (%)			*P* value^a^
	All (n = 312 145)	Residence in persistently impoverished CTs (n = 20 007)	No residence in persistently impoverished CTs (n = 292 138)	
Age, mean (SD), y	61.9 (13.3)	61.8 (13.3)	61.9 (13.3)	.75
Race				
Black	38 323 (12.3)	8735 (43.7)	29 588 (10.1)	<.001
White	245 257 (78.6)	10 413 (52.0)	234 844 (80.4)	
Other^b^	28 565 (9.2)	859 (4.3)	27 706 (9.5)	
Ethnicity				
Hispanic	26 397 (8.5)	2605 (13.0)	23 792 (8.1)	<.001
Non-Hispanic	285 748 (91.5)	17 402 (87.0)	268 346 (91.9)	
Marital status				
Married/partner	182 700 (58.5)	7886 (39.4)	174 814 (59.8)	<.001
Single	38 264 (12.3)	3370 (16.8)	34 894 (11.9)	
Separated/divorced	46 175 (14.8)	5154 (25.8)	41 021 (14.0)	
Widowed	45 006 (14.4)	3597 (18.0)	41 409 (14.2)	
Year of diagnosis				
2010	31 597 (10.1)	2094 (10.5)	29 503 (10.1)	<.001
2011	33 012 (10.6)	2135 (10.7)	30 877 (10.6)	
2012	33 583 (10.8)	2239 (11.2)	31 344 (10.7)	
2013	34 910 (11.2)	2336 (11.7)	32 574 (11.2)	
2014	35 238 (11.3)	2240 (11.2)	32 998 (11.3)	
2015	36 699 (11.8)	2392 (12.0)	34 307 (11.7)	
2016	37 253 (11.9)	2375 (11.9)	34 878 (11.9)	
2017	37 599 (12.0)	2320 (11.6)	35 279 (12.1)	
2018	32 254 (10.3)	1876 (9.4)	30 378 (10.4)	
RUCA				
Rural	31 487 (10.1)	5358 (26.8)	26 129 (8.9)	<.001
Urban	280 658 (89.9)	14 649 (73.2)	266 009 (91.1)	
Pathologic grade				
1	75 985 (24.3)	4137 (20.7)	71 848 (24.6)	<.001
2	143 186 (45.9)	8505 (42.5)	134 681 (46.1)	
3	92 974 (29.8)	7365 (36.9)	85 609 (29.3)	
Breast tumor subtype				
Luminal B (HR^+^, *ERBB2*^+^)	30 419 (9.7)	2041 (10.2)	28 378 (9.7)	<.001
Luminal A (HR^+^, *ERBB2*^−^)	237 419 (76.1)	13 895 (69.5)	223 524 (76.5)	
HER2 enriched (HR^−^, *ERBB2*^+^)	11 687 (3.7)	947 (4.7)	10 740 (3.7)	
Triple negative (HR^−^, *ERBB2*^−^)	32 620 (10.5)	3124 (15.6)	29 496 (10.1)	
AJCC staging				
I	182 855 (58.6)	10 215 (51.1)	172 640 (59.1)	<.001
II	99 121 (31.8)	7180 (35.9)	91 941 (31.5)	
III	30 169 (9.7)	2612 (13.1)	27 557 (9.4)	
SEER summary stage				
Localized	223 666 (71.7)	13 295 (66.5)	210 371 (72.0)	<.001
Regional	88 479 (28.3)	6712 (33.5)	81 767 (28.0)	
Chemotherapy				
No/unknown	195 478 (62.6)	11 503 (57.5)	183 975 (63.0)	<.001
Yes	116 667 (37.4)	8504 (42.5)	108 163 (37.0)	
Radiation				
No/unknown	131 955 (42.3)	9496 (47.5)	122 459 (41.9)	<.001
Yes	174 158 (55.8)	10 104 (50.5)	164 054 (56.2)	
Refused	6032 (1.9)	407 (2.0)	5625 (1.9)	
Breast surgery				
None	8502 (2.7)	815 (4.1)	7687 (2.6)	<.001
Lumpectomy	178 496 (57.2)	10 179 (50.9)	168 317 (57.6)	
Mastectomy	125 147 (40.1)	9013 (45.0)	116 134 (39.8)	
Axillary surgery				
None	26 767 (8.6)	1867 (9.3)	24 900 (8.5)	<.001
SLNB	236 443 (75.7)	13 821 (69.1)	222 622 (76.2)	
ALND	48 935 (15.7)	4319 (21.6)	44 616 (15.3)	
Reconstruction				
No	135 249 (43.3)	10 590 (52.9)	124 659 (42.7)	<.001
Yes	168 394 (53.9)	8602 (43.0)	159 792 (54.7)	
Unknown	8502 (2.7)	815 (4.1)	7687 (2.6)	
All-cause mortality	34 789 (11.1)	3253 (16.3)	31 536 (10.8)	<.001
Breast cancer mortality	14 880 (4.8)	1509 (7.5)	13 371 (4.6)	<.001

Abbreviations: AJCC, American Joint Committee on Cancer; ALND, axillary lymph node dissection; CT, census tract; *ERBB2* (formerly HER2), human epidermal growth factor 2; HR, hormone receptor (for either estrogen or progesterone); RUCA, rural-urban commuting area; SEER, Surveillance, Epidemiology, and End Results; SLNB, sentinel lymph node biopsy.

^a^ *P* values calculated using *t* test for continuous variables and χ^2^ tests for categorical variables.

^b^ The Other category includes patients identified as American Indian or Alaska Native, Asian or Pacific Islander, and Other, collapsed due to small sample sizes.

### Survival Outcomes

**Figure 1.**
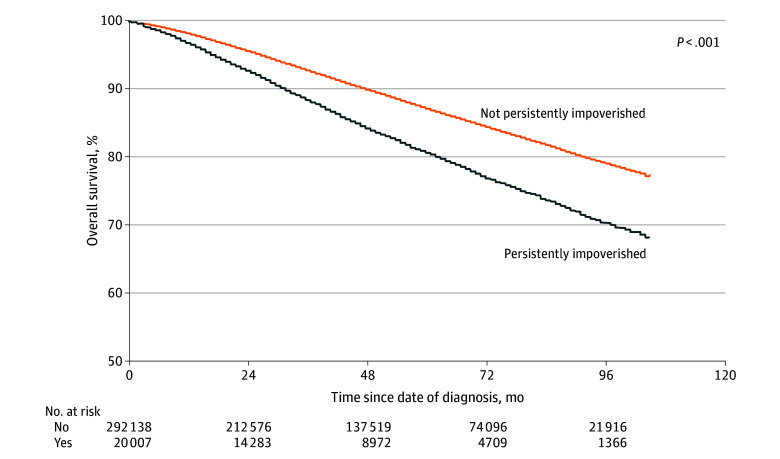
Overall Survival by Residence in Census Tracts With and Without Persistent Poverty (N = 312 145)

**Figure 2.**
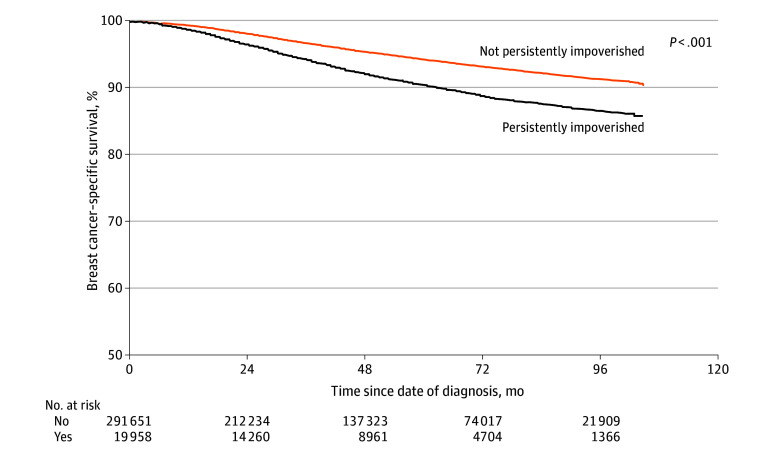
Breast Cancer–Specific Survival by Residence in Census Tracts With and Without Persistent Poverty (n = 311 609)

Patients living in persistently impoverished CTs had a 56% higher risk of all-cause mortality (HR, 1.56; 95% CI, 1.50-1.62) compared with individuals living in CTs without persistent poverty, which remained significant on multivariable analysis (AHR, 1.13; 95% CI, 1.08-1.18) (Table 2). Similarly, patients living in persistently impoverished areas had a higher risk of breast cancer–specific mortality on both univariable (HR, 1.70; 95% CI, 1.61-1.80) and multivariable (AHR, 1.10; 95% CI, 1.03-1.17) analysis (Table 2) compared with patients living in CTs that were not persistently impoverished. All-cause mortality rates (eTable 1 in Supplement 1) and breast cancer–specific mortality rates (eTable 2 in Supplement 1) diverged as early as 3 years following diagnosis (all-cause: RR, 1.62; 95% CI, 1.56-1.70; breast cancer–specific: RR, 1.80; 95% CI, 1.68-1.92). Mortality gaps widened as months since diagnosis progressed for both all-cause (Figure 1) and breast cancer–specific (Figure 2) mortality.

**Table 2.** Association Between Residence in a Persistently Impoverished Census Tract and All-Cause Mortality

Mortality type	Hazard ratio (95% CI)	
	Crude	Adjusted^a^
All-cause mortality (N = 312 145)	1.56 (1.50-1.62)	1.13 (1.08-1.18)
Breast cancer-specific mortality (n = 311 609)	1.70 (1.61-1.80)	1.10 (1.03-1.17)

^a^ Adjusted for age; race and ethnicity; marital status; Rural Urban Commuting Area code; Surveillance, Epidemiology, and End Results Program stage; pathologic grade; hormone receptor status; chemotherapy; radiotherapy; and breast and axillary surgery.

### Surgical Treatment

Regardless of persistent poverty status, use of lumpectomy increased from 2010 to 2018, whereas use of mastectomy decreased (eFigure 2 in Supplement 1). However, patients living in persistently impoverished areas were consistently more likely to undergo a mastectomy rather than lumpectomy. Similarly, rates of SLNB increased, whereas performance of ALND decreased over time, but patients living in areas with persistent poverty were more likely to undergo ALND vs SLNB throughout the study period (eFigure 3 in Supplement 1). Although overall use of breast reconstruction increased over time, patients residing in persistently impoverished areas remained consistently less likely to receive reconstruction (eFigure 4 in Supplement 1).

## Discussion

Patients in this cohort with stage I to III breast cancer living in persistently impoverished neighborhoods were more likely to identify with historically marginalized and minoritized populations (ie, Black race or Hispanic ethnicity), live in more rural regions, and present with more-aggressive tumor characteristics, including higher grade, molecular subtypes with worse prognostication, and greater disease burden. Although surgical management remained similar between groups, patients residing in persistently impoverished areas were consistently more likely to undergo ALND. Furthermore, patients living in persistently impoverished areas undergoing mastectomy were less likely to undergo breast reconstruction. A larger proportion of patients residing in areas with persistent poverty had a higher likelihood of receiving chemotherapy. Both all-cause and breast cancer–specific mortality risk remained higher among those living in CTs with persistent poverty despite receiving more aggressive treatments.

The role of persistent poverty in cancer outcomes has only recently gained attention in the literature, and our findings are consistent with existing research.^20^ Moss et al^10^ noted that patients living in counties with persistent poverty were disproportionately non-Hispanic Black and Hispanic, with a 1.6 per 100 000 person-year increase in cancer mortality rate compared with currently impoverished (but not persistently impoverished) counties, following adjustment of sociodemographic factors. Similarly, Papageorge et al^12^ noted that a higher proportion of patients living in persistent poverty were categorized as non-Hispanic Black and less likely to have HR-positive disease. Patients living in persistently impoverished counties were also more likely to undergo a radical mastectomy with higher risk of cancer-specific mortality after adjusting for demographic characteristics, molecular subtype, and stage of disease. Papageorge et al^12^ only included patients with breast cancers diagnosed between 2012 and 2016 and evaluated persistent poverty at a county level, which inherently leads to a larger degree of imprecision with both overinclusion and underinclusion of targeted areas.^21^ Their study also only evaluated breast surgery without consideration of axillary or reconstructive surgery or receipt of chemotherapy or radiotherapy, all of which have major implications on survival.^22^ The present study noted that patients living in persistently impoverished CTs had increased risks of all-cause and breast cancer–specific mortality despite controlling for all clinical and treatment factors, suggesting the likely presence of an additional distinct and likely nuanced mechanistic relationship associating persistent poverty with mortality beyond treatment receipt.

We also noted widening mortality gaps for both all-cause and breast cancer–specific mortality based on persistent poverty status, despite similar patterns and stable differences in surgical treatment over time. Moss et al^11^ similarly reported widening health disparities for mortality outcomes, particularly for Black patients living in persistently impoverished rural counties. While the cause of this expanding gap requires further investigation, the ever-growing wealth gap between the wealthiest and poorest families in the US has more than doubled between 1989 and 2016.^23^ Prior studies have established an association between relative poverty (rather than absolute poverty) and health outcomes, where counties with higher levels of relative income inequality experience greater cancer mortality.^24^ Greater income inequality has been hypothesized to encourage “resentment…hopelessness, and alienation,” leading to a “sense of injustice, discontent, and distrust” that erodes social cohesion.^25^ Social buffers (eg, social networks) disintegrate, which leads to withdrawal and disorganization of community structure and departure of businesses with loss of job opportunities, all of which fuel a cycle of declining social capital and further disinvestment.^25^ Historical institutional policies and private practices, such as the Jim Crow laws, which enforced residential racial segregation and differential resource allocation in the South, where most CTs with persistent poverty are located, further concentrated areas with poverty such that residents must cope with the consequences of both their own lack of income and lack of neighborhood income (eg, lack of labor force role models, high education dropout rates, exposure to unsupervised peer group activity).^8,25^ Moreover, the structural forces that contribute to the creation of persistent poverty limit any plausibility of upward economic mobility, particularly among low-income Black families, which only perpetuates intergenerational poverty.^21,26,27^ In rural areas, where there is already a shortage of health care personnel and supplies, lower insurance coverage, and longer distance to health care facilities, the collapsed infrastructure in persistently impoverished areas further restricts access to healthy food, recreational facilities, and healthy behaviors.^28^

The mechanism in which living in persistent poverty is associated with increased mortality risk warrants further evaluation. One plausible mechanism involves the chronic activation of the hypothalamic-pituitary-adrenal axis and the sympathetic-adrenal-medullary pathway, often measured through allostatic load.^29^ Prolonged exposure to adverse socioenvironmental stressors, such as neighborhood deprivation and racialized segregation, has been associated with high allostatic load burden.^30,31^ Studies have also evaluated associations between allostatic load with childhood and adult socioeconomic status and the impact of upward economic mobility.^32,33^ Existing studies have noted associations between allostatic load, neighborhood level racialized economic segregation, and mortality among patients with cancer.^34,35,36^ However, studies evaluating the role of persistent poverty on allostatic load are needed.

### Limitations

Interpretation of our findings is not without limitations. We are limited by the information SEER provides, such as the inability to distinguish between individuals who did not receive chemotherapy and/or radiotherapy from those for whom this information is unknown, which may inadvertently lead to misclassification bias. Comorbidity data are also not available in SEER, limiting our ability to control for the role of comorbidities in all-cause mortality. Persistent poverty was depicted through CTs, which better aligns with the association between neighborhoods and individual outcomes.^21^ However, CTs lack administrative boundaries and are too small and numerous for targeting economic development–related interventions.^21^ Additionally, complete geographic addresses are not always available for geocoding residential CTs, which may limit the quality of the persistent poverty measure. Individual addresses at the time of diagnosis similarly do not account for population mobility and residential history. Focusing on persistent poverty may also underestimate and not fully capture the multidimensional nature of the barriers that families living in persistently disadvantaged areas may experience.^37^ Nevertheless, the findings from our study add to the literature advocating for the necessity to advance equity for patients residing in persistently impoverished areas.

## Conclusions

The findings of this cohort study suggest that patients with nonmetastatic breast cancer who live in persistently impoverished CTs exhibit more-aggressive tumor characteristics and receive more-invasive surgeries compared with individuals residing in neighborhoods that are not persistently impoverished. However, these impoverished individuals have higher breast cancer–specific and all-cause mortality.

## Notes

Supplement 1. **eFigure 1.** Study Schema Showing Patient Inclusion/Exclusion Criteria **eTable 1.** Crude Incidence, Rate Difference and Risk Ratio of Mortality At 3, 6, and 9 Years Between Patients Living in Persistently Impoverished Census Tracts vs Patients Living in Non-Persistently Impoverished Census Tracts **eTable 2.** Crude Incidence, Rate Difference and Risk Ratio of Breast Cancer-Specific Mortality at 3, 6, and 9 Years Between Patients Living in Persistently Impoverished Census Tracts vs Patients Living in Non-Persistently Impoverished Census Tracts **eFigure 2.** Breast Surgery by Residence in Persistently Impoverished Census Tracts Over Time **eFigure 3**. Axillary Surgery by Residence in Persistently Impoverished Census Tracts Over Time **eFigure 4**. Reconstruction Surgery by Residence in Persistently Impoverished Census Tracts Over Time

Supplement 2.
Data Sharing Statement
