# Supplementary material for: Persistent Neighborhood Poverty and Breast Cancer Outcomes
Source: JAMA Netw Open. 2024 Aug 29;7(8):e2427755. doi: 10.1001/jamanetworkopen.2024.27755 (PMC11362869; doi:10.1001/jamanetworkopen.2024.27755)
Supplement: Supplement 1. — eFigure 1. Study Schema Showing Patient Inclusion/Exclusion Criteria eTable 1. Crude Incidence, Rate Difference and Risk Ratio of Mortality At 3, 6, and 9 Years Between Patients Living in Persistently Impoverished Census Tracts vs Patients Living in Non-Persistently Impoverished Census Tracts eTable 2. Crude Incidence, Rate Difference and Risk Ratio of Breast Cancer-Specific Mortality at 3, 6, and 9 Years Between Patients Living in Persistently Impoverished Census Tracts vs Patients Living in Non-Persistently Impoverished Census Tracts eFigure 2. Breast Surgery by Residence in Persistently Impoverished Census Tracts Over Time eFigure 3. Axillary Surgery by Residence in Persistently Impoverished Census Tracts Over Time eFigure 4. Reconstruction Surgery by Residence in Persistently Impoverished Census Tracts Over Time [file jamanetwopen-e2427755-s001.pdf]

Supplementary Online Content

Chen JC, Handley D, Elsaid MI, et al. Persistent neighborhood poverty and breast cancer outcomes. *JAMA Netw Open*. 2024;7(8):e2427755.
doi:10.1001/jamanetworkopen.2024.27755

eFigure 1. Study Schema Showing Patient Inclusion/Exclusion Criteria

eTable 1. Crude Incidence, Rate Difference and Risk Ratio of Mortality At 3, 6, and 9 Years Between Patients Living in Persistently Impoverished Census Tracts vs Patients Living in Non-Persistently Impoverished Census Tracts

eTable 2. Crude Incidence, Rate Difference and Risk Ratio of Breast Cancer-Specific Mortality at 3, 6, and 9 Years Between Patients Living in Persistently Impoverished Census Tracts vs Patients Living in Non-Persistently Impoverished Census Tracts

eFigure 2. Breast Surgery by Residence in Persistently Impoverished Census Tracts Over Time

eFigure 3. Axillary Surgery by Residence in Persistently Impoverished Census Tracts Over Time.

eFigure 4. Reconstruction Surgery by Residence in Persistently Impoverished Census Tracts Over Time

This supplementary material has been provided by the authors to give readers additional information about their work.

© 2024 Chen JC et al. *JAMA Network Open.*

eFigure 1. Study schema showing patient inclusion/exclusion criteria

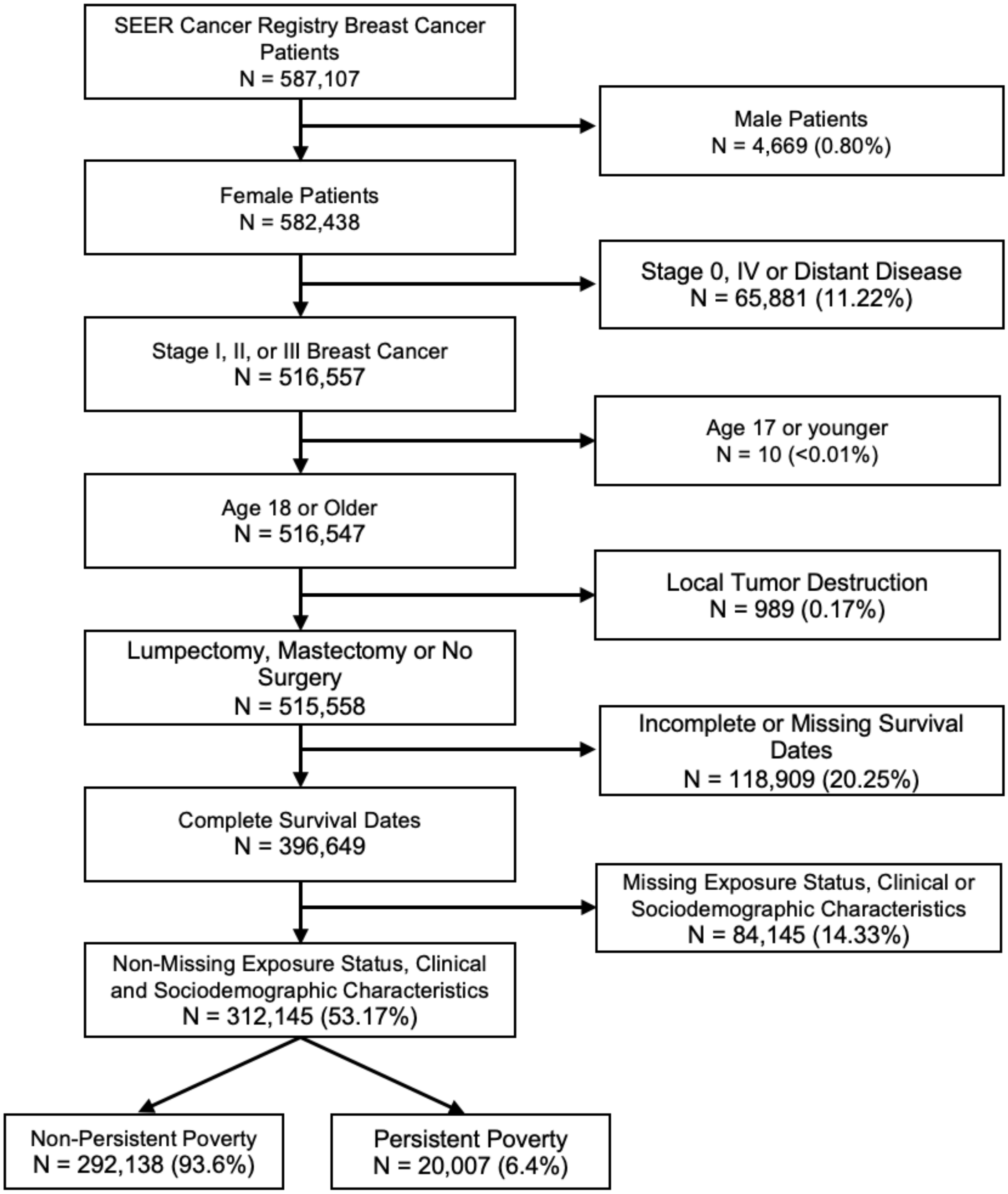

© 2024 Chen JC et al. *JAMA Network Open.*

Supplemental eTable 1. Crude incidence, rate difference and risk ratio of mortality at 3, 6, and 9 years between patients living in persistently impoverished census tracts vs patients living in non-persistently impoverished census tracts

Outcome	No. of participants	No. of events	Person-years	Mortality rate 100 person-years (95% CI)	Rate difference 100 person-years (95% CI)	Risk Ratio (95% CI)
**3-year mortality**						
Not persistently impoverished	292138	16906	694472.3	2.43 (2.40 to 2.47)	Reference	Reference
Persistently impoverished	20007	1885	46942.8	4.02 (3.84 to 4.20)	1.58 (1.40 to 1.77)	1.62 (1.56 to 1.70)
**6-year mortality**						
Not persistently impoverished	292138	28199	1055851.5	2.67 (2.64 to 2.70)	Reference	Reference
Persistently impoverished	20007	2973	70490.7	4.22 (4.07 to 4.37)	1.55 (1.39 to 1.70)	1.54 (1.49 to 1.59)
**9-year mortality**						
Not persistently impoverished	292138	31536	1158162.5	2.72 (2.69 to 2.75)	Reference	Reference
Persistently impoverished	20007	3253	76953.7	4.23 (4.08 to 4.38)	1.50 (1.36 to 1.65)	1.51 (1.46 to 1.56)

© 2024 Chen JC et al. *JAMA Network Open.*

Supplemental eTable 2. Crude incidence, rate difference and risk ratio of breast cancer-specific mortality at 3, 6, and 9 years between patients living in persistently impoverished census tracts vs patients living in non-persistently impoverished census tracts

Outcome	No. of participants	No. of events	Person-years	Mortality rate 100 person-years (95% CI)	Rate difference 100 person-years (95% CI)	Risk Ratio (95% CI)
**3-year mortality**						
Not persistently impoverished	291651	7823	693339.7	1.13 (1.10 to 1.15)	Reference	Reference
Persistently impoverished	19958	961	46852.6	2.05 (1.92 to 2.19)	0.92 (0.79 to 1.06)	1.80 (1.68 to 1.92)
**6-year mortality**						
Not persistently impoverished	291651	12335	1054242.2	1.17 (1.15 to 1.19)	Reference	Reference
Persistently impoverished	19958	1429	70371.7	2.03 (1.93 to 2.14)	0.86 (0.75 to 0.97)	1.69 (1.61 to 1.79)
**9-year mortality**						
Not persistently impoverished	291651	13371	1156485.1	1.16 (1.14 to 1.18)	Reference	Reference
Persistently impoverished	19958	1509	76828.8	1.96 (1.87 to 2.07)	0.81 (0.71 to 0.91)	1.65 (1.57 to 1.74)

© 2024 Chen JC et al. *JAMA Network Open.*

eFigure 2. Breast surgery by residence in persistently impoverished census tracts over time.

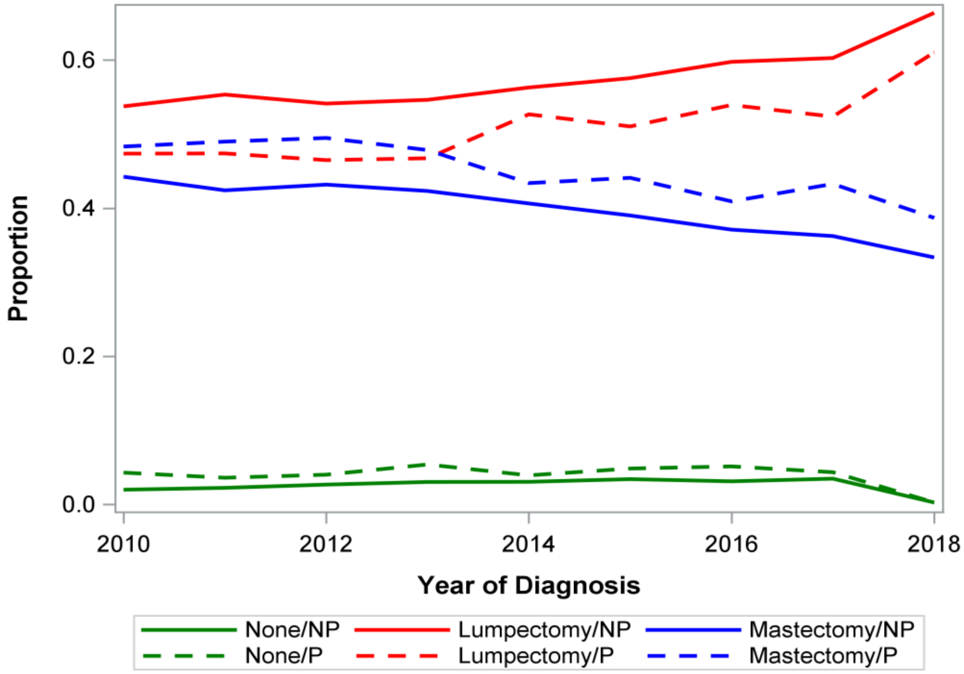

Solid line = Non-poverty; Dashed line = Poverty
Green = No surgery; Red = Lumpectomy; Blue = Mastectomy

© 2024 Chen JC et al. *JAMA Network Open.*

eFigure 3. Axillary surgery by residence in persistently impoverished census tracts over time.

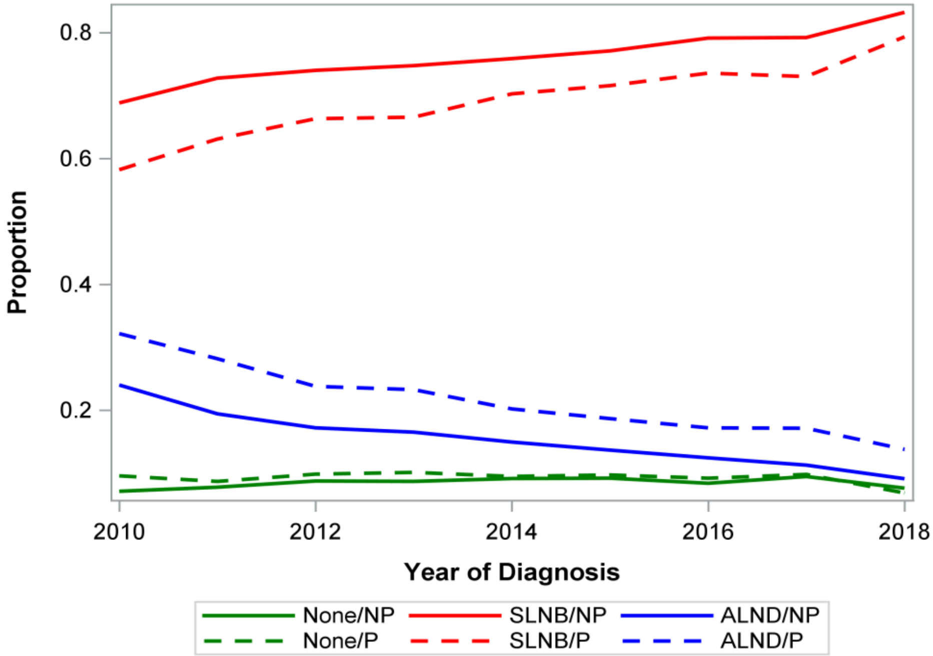

Solid line = Non-poverty; Dashed line = Poverty
Green = No surgery; Red = SLNB; Blue = ALND

© 2024 Chen JC et al. *JAMA Network Open.*

Supplementary eFigure 4. Reconstruction surgery by residence in persistently impoverished census tracts over time.

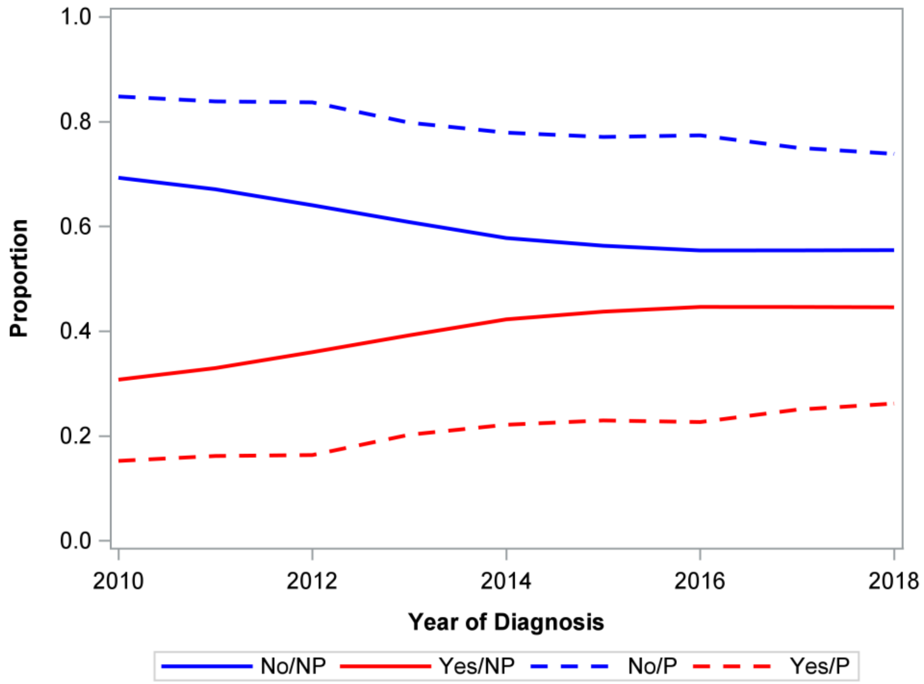

Solid line = Non-poverty; Dashed line = Poverty
Red = Yes; Blue = No

© 2024 Chen JC et al. *JAMA Network Open.*
