# Supplementary material for: Persistent Neighborhood Poverty and Breast Cancer Outcomes
Source: JAMA Netw Open. 2024 Aug 29;7(8):e2427755. doi: 10.1001/jamanetworkopen.2024.27755 (PMC11362869; doi:10.1001/jamanetworkopen.2024.27755)
Supplement: Supplement 2. — Data Sharing Statement [file jamanetwopen-e2427755-s002.pdf]

Data Sharing Statement

Chen. Persistent Neighborhood Poverty and Breast Cancer Outcomes. *JAMA Netw Open*. Published August 29, 2024. doi:10.1001/jamanetworkopen.2024.27755

Data
Data available: No

Additional Information
Explanation for why data not available: Data was obtained from a public database (SEER)
